# Whole genome identification and operational analysis of the *bHLH* gene family in longan and the role of *DlbHLH8* in flowering regulation

**DOI:** 10.3389/fpls.2026.1824280

**Published:** 2026-06-10

**Authors:** Yao Liu, Xuelian Sang, Liqin Liu, Ci Ren, Zhixin Zhang, Tao Chen, Hongli Li, Dengwei Jue, Shengyou Shi

**Affiliations:** 1 Chongqing Key Laboratory for Germplasm Innovation of Special Aromatic Spice Plants, College of Smart Agriculture/Institute of Special Plants, Chongqing University of Arts and Sciences, Yongchuan, China; 2 National Key Laboratory for Tropical Crop Breeding, Institute of Tropical Crop Genetic Resources, Chinese Academy of Tropical Agricultural Sciences, Haikou, China; 3 Horticulture Research Institute, Guangxi Academy of Agriculture Sciences, Nanning, China; 4 Tropical Crops Genetic Resources Institute, Chinese Academy of Tropical Agricultural Sciences, Haikou, China

**Keywords:** longan, bHLH gene family, DlbHLH8 gene, flower development regulation, plant physiology

## Abstract

**Background:**

Longan (*Dimocarpus longan* Lour.) is an important tropical and subtropical fruiting crop; understanding its flower formation regulatory mechanisms represents an important direction in floral growth and evolution research. The basic helix-loop-helix (bHLH) transcription factor family plays crucial roles in diverse physiological processes in plants, including growth, development, and stress responses. However, the roles of *bHLH* genes in longan flower development have not been investigated.

**Results:**

We detected 126 *bHLH* family members in the longan genome database. The *bHLH* genes of longan and *Arabidopsis* were further classified into 27 subfamilies. Structural analysis indicated that DlbHLH proteins were comparatively well-conserved within specific subfamilies. The results of collinearity analysis, supported by Ka/Ks (Non-synonymous substitution rate/Synonymous substitution rate) analysis, indicated that the expansion of the *DlbHLH* gene family was primarily driven by segmental duplication followed by strong purifying selection. Examination of the promoter regions of the *DlbHLH* gene family suggested that *DlbHLH* participated in the control of floral bud development. *bHLH* family genes exhibited diverse distinct expression profiles during the flowering processes of the varieties “Shixia” and “Sijimi” (SJ), with *DlbHLH8* exhibiting a dynamic and cultivar-specific expression pattern, including a 7.49-fold reduction in expression during the critical T1-T2 phase of flower development in the SJ variety. Phenotypic observations revealed that under long-day circumstances, genetically modified *Arabidopsis* plants overexpressing *DlbHLH8* exhibited significantly earlier flowering than wild-type plants. Gene expression analysis showed that *DlbHLH8* overexpression was associated with the altered expression of key genes in the flowering pathway, namely *AtAP1* (*Arabidopsis thaliana APETALA1*), *AtLFY* (*Arabidopsis thaliana LEAFY*), and *AtFLC* (*Arabidopsis thaliana FLOWERING LOCUS C*). These findings offer a basis for additional research into the regulatory mechanisms of bHLH proteins in longan flower development.

**Conclusion:**

Some *DlbHLH* family members play important roles in the regulation of plant flowering; *DlbHLH8* may promote plant flowering in longan by regulating *AtAP1*, *AtLFY*, and *AtFLC*.

## 1 Introduction

Longan is widely cultivated in Southeast Asia and other tropical regions. Longan fruit is rich in nutritional components, such as sugar, vitamin C, various amino acids, and minerals, and possesses medicinal value, making it an economically important crop (Zhang et al., 2020). However, the flower formation process of longan is complex and highly sensitive to photoperiod; the flowering time directly determines the yield and quality of the fruit. In actual production, chemical reagents (e.g., potassium chlorate) are utilized to force out-of-season flowering in the “Shixia” (SX) longan cultivar (Zhao et al., 2026). However, this induction may negatively affect the quality of certain longan varieties. For example, in a previous study fruit quality was adversely affected by the application of potassium chlorate to the ‘Guiguanzao’ variety, notwithstanding the promotion of flower formation, reduced fruit drop rate, and increased yield. Moreover, although the application of potassium chlorate in early December delayed the flowering of longan trees during the main season, this effect was not significant. Therefore, conducting in-depth genomic-level analyses to elucidate the intrinsic molecular mechanisms regulating flowering in longan trees (Lithanatudom et al., 2025), with the goal of selecting and breeding new varieties of early- and late-blooming longan and improving the yield and quality of longan fruit in large-scale production, is necessary.

Transcription factors play a central role in the complex floral developmental regulatory networks of plants. The *bHLH* (basic Helix-Loop-Helix) gene family is a class of transcription factors widely present in eukaryotes (Corinna and Boas, 2023). The bHLH transcription factor is characterized by the presence of a highly conserved bHLH domain comprising approximately 60 amino acids (Radani et al., 2023). The N-terminal domain of this structure is a region rich in basic amino acids responsible for recognizing and binding specific E-box sequences on DNA, whereas the C-terminal domain is formed by two amphipathic α-helices connected via a variable loop region, creating a helix-loop-helix structure. This structure mediates homologous or heterologous dimerization among bHLH proteins and precisely regulates gene transcription by modulating cis-acting elements such as the E-box (CANNTG) in the promoter region of downstream target genes (Michael et al., 2023). The number of *bHLH* members varies among plants; for example, 309 members have been identified in bamboo (*Dendrocalamus latiflorus*) (Zeng et al., 2024), whereas 110 have been identified in *Vitis vinifera* (Wang et al., 2018). *bHLH* members play key roles in the response of the plant to abiotic stresses, such as drought, high salinity, low temperature, and nutrient deficiency. Wang et al. demonstrated that the overexpression of *MfbHLH38* in *Arabidopsis* significantly enhanced drought tolerance in transgenic plants by modulating the ABA signaling pathway and reactive oxygen species elimination systems (Qiu et al., 2020). Zhou et al. discovered that the PtrbHLH66 protein in poplars could directly attach to the promoter of the *PtrCBF* gene, positively regulating its expression and thereby enhancing cold resistance (Liang et al., 2022). Under biotic stress, Liu et al. *Ralstonia solanacearum* induced the expression of the *CabHLH79* gene in chili peppers. After reducing the expression of the *CabHLH79* gene via virus-triggered gene silencing, the susceptibility of peppers to bacterial wilt increased, indicating that *CabHLH79* played a positive regulatory role in disease resistance in chili peppers (Wang et al., 2022).

The *bHLH* family exhibits significant functional diversity and species specificity across different plants. In *Chimonobambusa utilis*, *CubHLH17* regulates the synthesis of red pigments in the sheath of bamboo culms, thereby affecting its ornamental and economic value (Tong et al., 2025). In poplars, *PtrbHLH186* regulates the expression of secondary cell wall cellulose synthase genes, which is crucial for wood cellulose synthesis (Liu et al., 2022). In apples, *MdbHLH16* and *MdbHLH33* activate the expression of anthocyanin synthesis genes, promoting the formation of apple peel (Xu H, et al., 2017). In pears, *PpbHLH64* is a key factor in the negative regulation of anthocyanin accumulation (Tao et al., 2020). The *bHLH* gene family exhibits distinct floral regulatory expression patterns. In *Dendrobium*, *DlbHLH* is expressed to a great degree during flower development (Zeng et al., 2024), whereas in grapes, *VvbHLH003* and *VvbHLH007* are specifically expressed in stamens and inflorescences and are speculated to participate in the formation of floral organs (Wang et al., 2018). The flowering habit of longan, a typical tropical and subtropical fruiting species, entails conserved photoperiod regulation modules, which may have evolved as an adaptive response to the local ecology and environment. However, to date, the *bHLH* gene family has not been systemically identified in longan; its functional role in the flowering transition has not been investigated.

Against this backdrop, we aimed to perform a genome-wide identification and bioinformatics analysis of the longan *bHLH* gene family, with an emphasis on the expression patterns and functions of its members during flower induction. This research fills a gap in the study of the longan *bHLH* gene family, providing new perspectives and critical gene resources for a profound comprehension of the molecular mechanisms controlling flower formation in longan. The results of this study also serve as a solid theoretical foundation for the molecular breeding and genetic improvement of longan trees.

## 2 Materials and methods

### 2.1 Identification of *DlbHLH* gene in longan

We downloaded longan genomic data from the longan database (http://www.sapindaceae.com/Download.html) and used the Hidden Markov Model program (Ma et al., 2025) and the related Pfam database (http://pfam.xfam.org/PF00010) (Paysan-Lafosse et al., 2025) to screen all *bHLH* genes in the longan genome. The screened *bHLHs* were re-identified using the SMART online software (http://smart.embl-heidelberg.de/) and NCBI CD (http://www.ncbi.nlm.nih.gov/Structure/cdd/wrpsb.cgi). To better distinguish the *DlbHLH* gene, we annotated it based on its physical location in the longan genome. The physicochemical properties of DlbHLH proteins, including the number of amino acids (aa), molecular weight (MW), theoretical isoelectric point (pI), and instability index (values < 40 indicate stability), were analyzed using the online tool ProtParam (http://web.expasy.org/protparam/). Lastly, the subcellular localization of *DlbHLH* members was predicted using WoLF PSORT (https://wolfpsort.hgc.jp/) (Azimi et al., 2024).

### 2.2 Construction of phylogenetic tree

We constructed a phylogenetic tree using longan and the model plant *Arabidopsis thaliana*. First, we downloaded *Arabidopsis* bHLH protein information from the *Arabidopsis* Information Resource Database (http://cucurbitgenomics.org/). MEGA was utilized to construct phylogenetic trees of the *bHLH* gene families in longan and *Arabidopsis thaliana* using the maximum likelihood (ML) method. Briefly, multiple sequence alignments of bHLH amino acid sequences were performed using the MUSCLE algorithm with default parameters. The aligned sequences were manually inspected and trimmed to remove poorly aligned regions. The best-fit evolutionary model for the ML analysis was determined to be the Jones-Taylor-Thornton model (JTT) model with a discrete Gamma distribution (+G) to model evolutionary rate differences among sites. The number of bootstrap repeat builds of the phylogenetic tree was set to 1,000. The results were visualized using iTOL (https://itol.embl.de/).

### 2.3 Genetic structure and protein sequence

TBtools v1.09876 software was used to extract the distribution information concerning introns and exons of the *DlbHLH* gene from the GFF3 file of the longan *bHLH* genome and visualize the results. The protein motifs (maximum 15) of all DlbHLH members were analyzed using the MEME online website (https://meme-suite.org/meme/tools/meme); the results were visualized.

### 2.4 Chromosome analysis and homology of *DlbHLH*

The physical chromosomal location of *DlbHLH* members was analyzed using TBtools. A homology analysis of the *DlbHLH* gene family was carried out to determine the relationships of *DlbHLH* members; the results were visualized. To investigate the selection pressure on segmentally duplicated *DlbHLH* gene pairs, non-synonymous (Ka) and synonymous (Ks) substitution rates were calculated using the Simple Ka/Ks Calculator (NG) implemented in TBtools. Gene pairs with evidence of synonymous site saturation (defined as pS ≥ 0.75, resulting in undefined Ks values) were excluded from further analysis to ensure the reliability of Ka/Ks estimates.

### 2.5 Collinearity analysis of *DlbHLH* genes

The whole-genome data of *Arabidopsis thaliana*, lychee, and longan were downloaded from the Ensembl Plants database (https://plants.ensembl.org/index.html); they were visualized and analyzed for collinearity using TBtools.

### 2.6 *DlbHLH* Cis-element analysis

The *DlbHLH* promoter sequence was retrieved from 2,000 bp upstream of the start codon of the gene using TBtools. The Plant CARE database (https://bioinformatics.psb.ugent.be/webtools/plantcare/html/) was used to predict the sequences of *DlbHLH* members using TBtools for visualization analysis.

### 2.7 General analysis of *DlbHLH* expression

We downloaded the general data of expression in different tissues of longan from the NCBI sequence read archive (GSE84467) and analyzed the expression pattern of the *DlbHLH* gene in the following tissues: root, stem, leaf, flower, fruit, peel, pulp, seed, leaf bud, and flower bud. This dataset included three biological replicates per tissue. Utilizing RNA-seq data from the Jue Dengwei research group in the early stage, expression patterns of the *DlbHLH* gene in the three flowering stages (T1, T2, and T3) of longan cultivars “Sijimi” (SJ) and SX were analyzed. For this developmental stage dataset, three biological replicates were collected for each stage and cultivar. All RNA-seq libraries were sequenced on the Illumina HiSeq platform. Read counts were normalized using the Fragments Per Kilobase of transcript per Million mapped reads method. Differential expression between stages was determined using the DESeq2 package with a threshold of |log2(Fold Change)| > 1 and false discovery rate < 0.05. Heat maps were drawn using TBtools (Jue et al., 2018a).

### 2.8 Subcellular localization analysis

The full-length coding sequence of the *DlbHLH* gene without stop codons was first PCR-amplified using primers SUDlbHLH-S and SUDlbHLH-A and subsequently cloned into the pBWA(V)HS-osgfp vector to construct a *35S* promoter–controlled DlbHLH8-GFP fusion expression vector. The GFP-empty vector (pBWA(V)HS-osgfp) was used as a control for nuclear localization (Kiselev et al., 2021). Subsequently, the target plasmid pBWA(V)HS-osgfp was successfully obtained by restriction endonuclease digestion of the pBWA(V)HS-DlbHLH8 plasmid, followed by recovery and ligation of the digestion products. The plasmid was transformed into *Escherichia coli* DH5α; following positive detection, appropriate strains for DNA sequencing were chosen. To perform subcellular localization observations, we used the empty vector pBMA(V)-HS-osgfp as a control; the above-mentioned plasmids were respectively introduced into *Arabidopsis thaliana* protoplasts by a PEG-mediated method and cultured in a dark environment for 24–48 h. Lastly, image analysis was performed using a Leica laser confocal microscope.

### 2.9 Obtaining transgenic *Arabidopsis* plants

The overexpression vector pBI121-DlbHLH8 was constructed by inserting the entire coding DNA sequence of *DlbHLH8* (the empty pBI121 vector was also transformed into wild-type plants as a negative control), which was regulated by the CaMV 35S promoter, into the BamHI and SacI restriction endonuclease sites of the pBI121 vector. After the carrier was introduced into *Agrobacterium* GV3101 by freeze-thawing, wild-type *Arabidopsis thaliana* was transformed by inflorescence impregnation. The transformed seeds were sown on MS (Murashige and Skoog medium) solid medium supplemented with 30 μg/mL hygromycin and grown under 24 °C with a light cycle of 16-h light/8-h dark to screen positive transgenic lines (Cao et al., 2025).

### 2.10 Phenotype of transgenic plants

T3-generation transgenic plants were grown together with wild-type *Arabidopsis* plants under long-day conditions (16-h light/8-h dark, light intensity 3,000 lx, and temperature 24 °C). For phenotypic analysis of flowering time and rosette leaf number, at least 15 individual plants were examined for each genotype (WT (Wild Type), OE (Overexpression) 5, and OE8). Leaf samples were collected before flowering in wild-type plants; flowering period and rosette leaf count were recorded to evaluate phenotypic differences in flowering. Total RNA was extracted from T3-generation plants using a Biosharp Polysaccharide Plant Total RNA Extraction Kit and reverse-transcribed into cDNA using All-in-One First-Strand Synthesis MasterMix (containing dsDNase, Jiangsu Bristol). The expression levels of flowering-related genes, namely *AtAP1*, *AtLFY*, and *AtFLC* (**Supplementary Table 1**), were analyzed using RT-qPCR with *AtTVB-2* as an internal reference gene. PCR primers were designed using Primer Premier 12 and synthesized by Shenggong Bioengineering Co., Ltd. (China). The RT-qPCR system was composed of 10 µL Taq SYBR Green Premix, 0.5 µL of each upstream and downstream primer, 1 µL cDNA, and 8 µL ddH_2_O. Real-time fluorescent quantitative PCR was performed using the qTOWER 2.2 instrument (Jena Analytical Instruments AG, Germany). The amplification reaction conditions were set as follows: 94 °C pre-denaturation for 2 min; 94 °C for 30 s; 60 °C for 30 s; and 72 °C for 30 s, followed by melting curve analysis after 35 cycles (Gou et al., 2025). Gene expression levels were quantified using the 2^−ΔΔCt^ method, with mean values computed via Excel. One-way ANOVA was conducted using SPSS to analyze changes in the expression of target genes across different tissues and materials, with a significance threshold of P < 0.05. For phenotypic data, statistical significance was determined using Student’s *t*-test. The significance is indicated in the Figure legends. The final data were processed and visualized as heat maps using TBtools.

## 3 Results

### 3.1 Identification of *bHLH* members and analysis of physicochemical properties

In total, 126 longan *bHLH* genes were identified using HMMER3.0 to compare and verify conserved domains through Pfam and SMART. The *bHLH* genes were renamed *bHLH1* to *bHLH126* based on their physical chromosomal localization (**Supplementary Table 2**). Using TBtools, we examined the physicochemical properties of 126 *DlbHLH* genes, including Molecular Weight (MW), Isoelectric point (pI), and the number of coding Amino Acid (AA). The MW ranged from 8.83 kDa (*DlbHLH53*) to 132.02 kDa (*DlbHLH74*), with an average of 39.96 kDa. The pI varied from 4.58 (*DlbHLH66*) to 11.4 (*DlbHLH53*), averaging 6.98. The length of the coding AA ranged from 76 (*DlbHLH53*) to 1,167 AA (*DlbHLH74*), with an average length of 361. Although the conserved domains of bHLH transcription factors contain basic regions, acidic AA are enriched in protein molecules; 59.52% of longan bHLH family proteins have a pI < 7, with most exhibiting weak acidity. The instability index was between 32.78 and 91.43, with 98.41% of proteins being unstable (II > 40). Only two proteins (DlbHLH52 and DlbHLH95) were stable (II < 40). The aliphatic index ranged from 49.59 to 113.22. The subcellular distribution of 126 bHLH proteins in longan was analyzed using the online software WoLF PSORT. The outcomes indicated that 85.71% of the bHLH proteins were distributed in the nucleus, whereas a small proportion of the proteins were distributed in the cytosol (DlbHLH12, DlbHLH81, DlbHLH84, etc.), chloroplasts (DlbHLH11, DlbHLH47, DlbHLH88, DlbHLH118), and mitochondria (DlbHLH53, DlbHLH78, DlbHLH120).

### 3.2 Chromosomal localization and distribution of longan *bHLH* members

**Figure 1:**
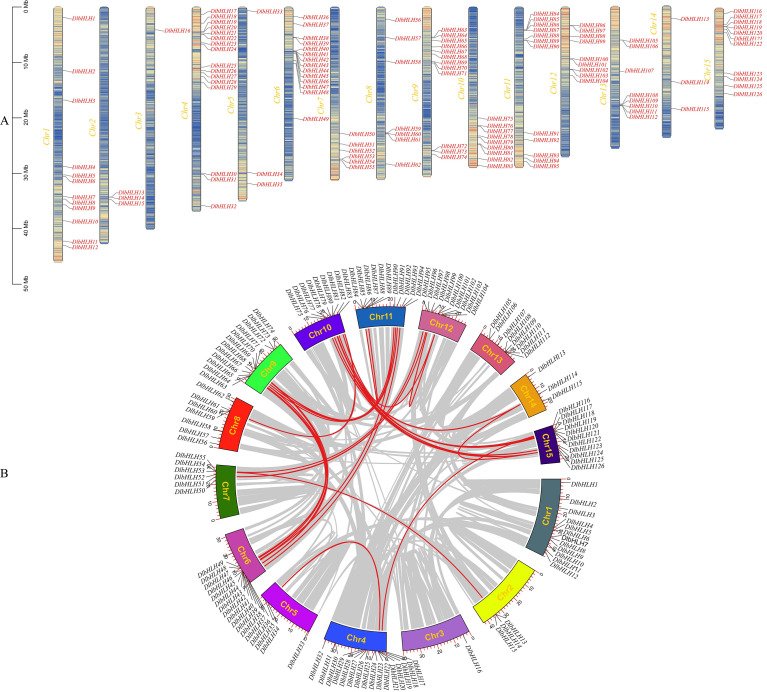
Chromosomal analysis of *DlbHLH* genes **(A)**. Collinearity analysis of *DlbHLH*: red lines denote collinear gene pairs; gray lines represent homologous blocks in the longan genome **(B)**.

Based on the results of physical chromosomal localization (**Figure 1A**), 126 *DlbHLH* genes were located on 15 chromosomes; however the distribution of these genes was uneven. Chromosome (Chr) 4 contained the highest number of *DlbHLH* genes (16 genes), followed by Chr 6 (14 genes). Chr 1 and Chr 9 each contained 12 different *DlbHLH* genes. Conversely, Chr 3 only had one gene (*DlbHLH16*). Additionally, 12 genes had seven tandem repeats and 26 segmental duplications involving *bHLH* genes (**Figure 1B**, **Supplementary Table 3**). These findings suggest that gene duplication is associated with the expansion of the *DlbHLH* family; these duplication events may act as the main driving force behind the evolution of the *DlbHLH* family.

We used the simple Ka/Ks Calculator (NG) in TBtools to analyze 26 segmental duplications involving *bHLH* genes. The results showed that pairs with synonymous site saturation (pS ≥ 0.75, resulting in undefined Ks values) were excluded from the calculation of Ka/Ks distribution statistics, leaving 23 (**Table 1**) high-confidence pairs for further analysis. The remaining gene pairs had Ka/Ks values < 1, indicating that strong purifying selection was the predominant evolutionary force acting on these duplicated genes post-divergence.

**Table 1:** Ka/Ks analysis of segmentally duplicated *DlbHLH* gene pairs.

Duplicate gene pair	Ka	Ks	Ka/Ks
*DlbHLH13/DlbHLH54*	0.51	1.63	0.31
*DlbHLH117/DlbHLH18*	0.37	1.89	0.20
*DlbHLH21/DlbHLH34*	0.28	1.67	0.17
*DlbHLH93/DlbHLH37*	0.48	1.56	0.31
*DlbHLH38/DlbHLH71*	0.35	1.85	0.19
*DlbHLH39/DlbHLH69*	0.26	2.70	0.10
*DlbHLH91/DlbHLH40*	0.48	2.36	0.20
*DlbHLH46/DlbHLH66*	0.27	1.59	0.17
*DlbHLH47/DlbHLH65*	0.58	1.66	0.35
*DlbHLH48/DlbHLH63*	0.35	2.24	0.16
*DlbHLH98/DlbHLH51*	0.35	2.07	0.17
*DlbHLH97/DlbHLH55*	0.29	1.57	0.18
*DlbHLH83/DlbHLH59*	0.50	1.35	0.37
*DlbHLH92/DlbHLH68*	0.43	1.85	0.23
*DlbHLH91/DlbHLH70*	0.43	1.50	0.29
*DlbHLH75/DlbHLH124*	0.36	1.62	0.22
*DlbHLH77/DlbHLH114*	0.48	1.86	0.26
*DlbHLH77/DlbHLH125*	0.31	1.71	0.18
*DlbHLH79/DlbHLH121*	0.28	1.35	0.21
*DlbHLH82/DlbHLH102*	0.49	2.18	0.23
*DlbHLH84/DlbHLH89*	0.31	0.43	0.72
*DlbHLH99/DlbHLH103*	0.28	1.22	0.23
*DlbHLH114/DlbHLH125*	0.41	1.70	0.24

### 3.3 Phylogenetic analysis of the longan *bHLH* gene family

**Figure 2:**
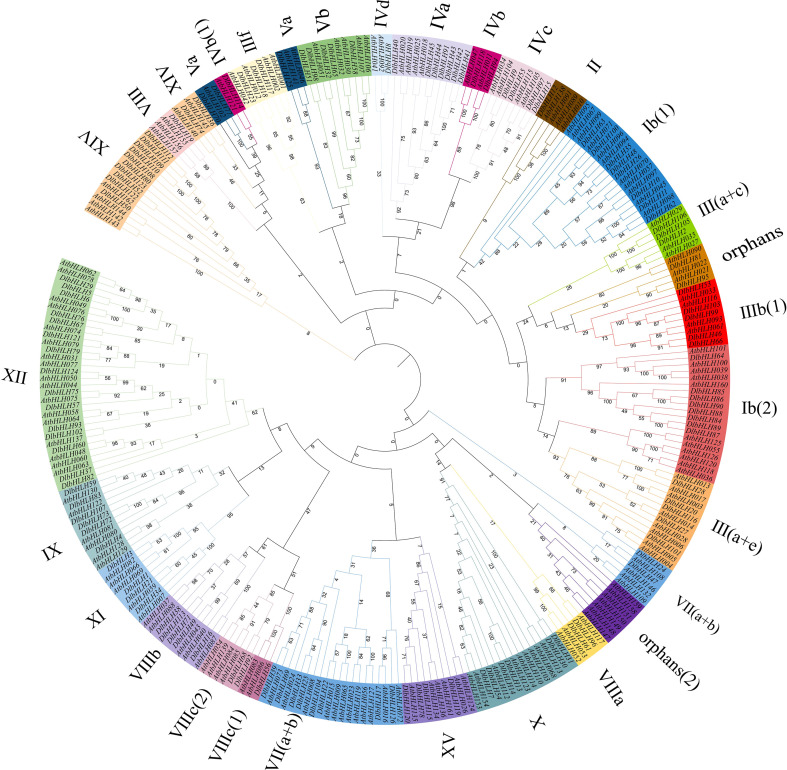
Phylogenetic tree of the relationships between *DlbHLH* and *AtbHLH*. Different subgroups are named according to *Arabidopsis* reports and distinguished by different colors.

To investigate the evolutionary relationships of the longan *bHLH* gene family, an phylogenetic tree was created using 126 identified longan bHLH protein sequences and 161 *Arabidopsis* bHLH protein sequences (**Figure 2**). The phylogenetic tree constructed using the Maximum Likelihood (ML) method showed that the *bHLH* gene family of *Arabidopsis* and longan was divided into 27 subgroups, among which the 126 *DlbHLH* genes were distributed across 26 of these subgroups. Notably, no longan *DlbHLH* member was found in the orphans(2) subfamily. The XII subfamily contained the highest number of *DlbHLH* members, totaling 15; the XIV subfamily followed with 10 members. The Ib(1), Ib(2), and VII(a+b) subfamilies each had eight *DlbHLH* members. The IVa subfamily had seven members; the IX and X subfamilies each contained six members. The VIIIb, III(a+e), III(1), and Vb subfamilies each had five members; the XI, VX, and IVc subfamilies each had four members. The III(a+c) and IIIf subfamilies each had three members; the VIII, IVb, IVd, and II subfamilies each had two members. The VIIIc(1), II, IVd, Va, IVd(1), and VIII subfamilies each had the fewest members, with only one *DlbHLH* member.

### 3.4 Analysis of conserved motifs and gene structures in the longan *bHLH* gene family

**Figure 3:**
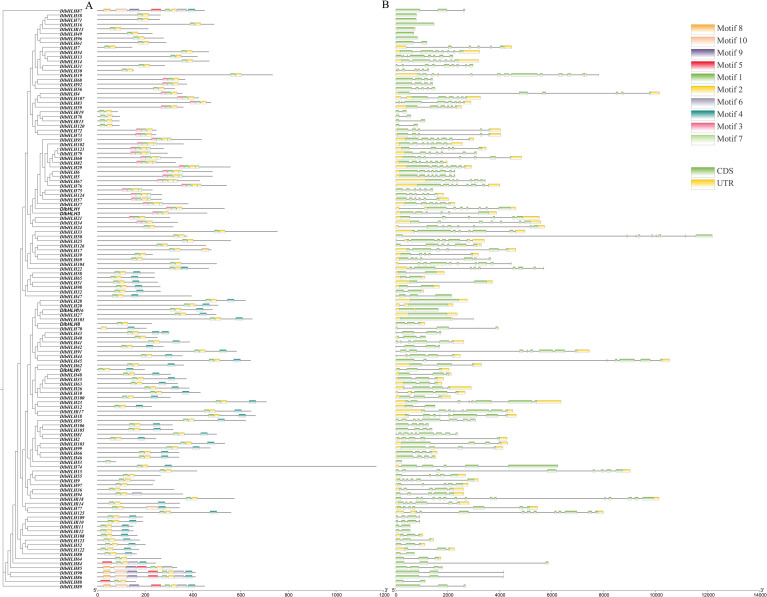
Distribution of conserved motifs **(A)** and gene structures **(B)** of *DlbHLH* genes.

Conserved motifs and domains of bHLH family proteins in the cultivar SX were systematically identified using the online MEME software and Conserved Domain Database (CDD) analysis using NCBI tools (**Figure 3**), with DlbHLH designated as one of the 10 conserved motifs (**Supplementary Tables 4**, **5**). Among the 126 bHLH protein sequences of longan, only DlbHLH70 lacked motif 1, whereas DlbHLH53 lacked motif 2. All other DlbHLH proteins contained both motifs (**Figure 3A**), indicating that motif 1 and motif 2 exhibited high conservation within the DlbHLH protein family. DlbHLH84, DlbHLH85, DlbHLH86, DlbHLH87, DlbHLH88, DlbHLH89, and DlbHLH90 shared motif 5, whereas the other DlbHLH proteins lacked it. Similarly, DlbHLH84, DlbHLH85, DlbHLH86, DlbHLH87, DlbHLH89, and DlbHLH90 shared motif 9; other DlbHLH proteins lacked it. Exon-intron structure analysis revealed that most DlbHLHs contained exons with numbers ranging from 1 to 14 (**Figure 3B**). However, 39 DlbHLHs lacked introns, although most DlbHLHs possessed 1–10 introns. DlbHLH117 possessed the highest number of exons and introns among all members, with 14 and 15, respectively.

### 3.5 Collinearity analysis of *DlbHLH* genes

**Figure 4:**
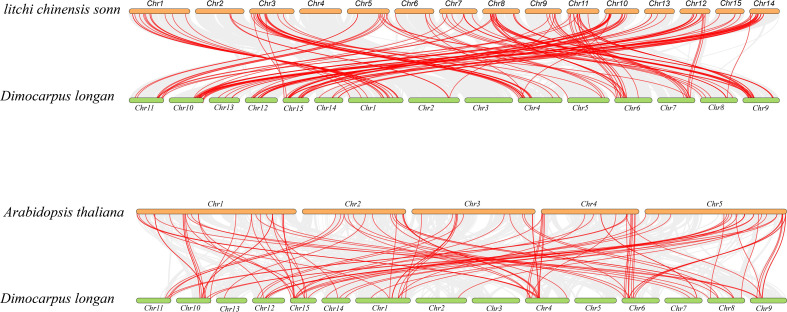
Collinearity relationships of *bHLH* genes between longan, lychee, and *Arabidopsis thaliana*, with the collinear distribution of *DlbHLH*. Red lines highlight collinear gene pairs, whereas gray lines indicate homologous segments in longan and other plant genomes.

To further explore the evolutionary connections between longan *bHLH* genes and those of other species, we conducted an interspecies collinearity analysis using longan, lychee, and *Arabidopsis thaliana* (**Figure 4**). In total, 152 pairs of orthologous *bHLH* genes were identified in longan and lychee; 105 pairs of orthologous *bHLH* genes were identified in longan and *Arabidopsis thaliana* (**Supplementary Table 6**). Therefore, the number of orthologous *bHLH* genes between longan and lychee was relatively high, indicating a close phylogenetic relationship. The number of collinear genes between longan and *Arabidopsis thaliana* was significantly fewer than that between longan and lychee. This finding revealed differences in their gene structure and function and pointed to the significant differentiation of their gene families during evolution.

### 3.6 Analysis of promoter Cis-elements in the *bHLH* gene family

**Figure 5:**
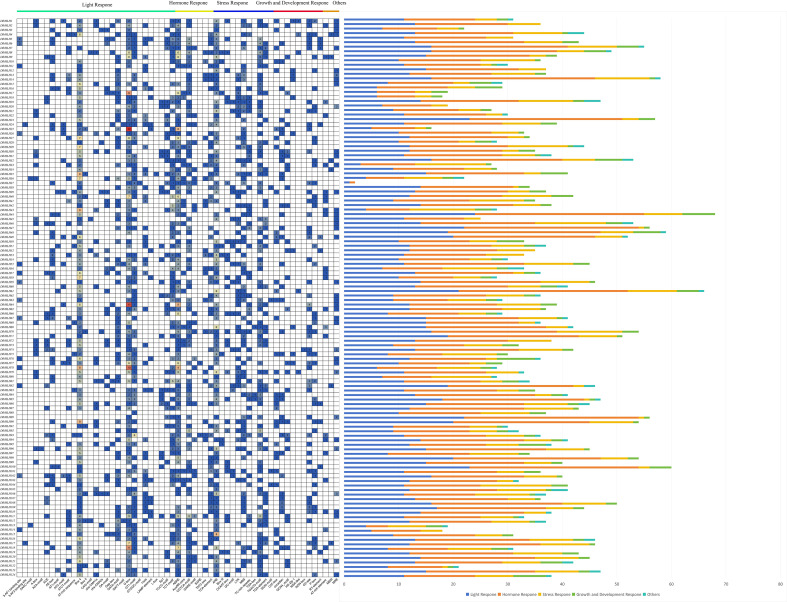
Distribution of cis-regulatory elements among 126 *DlbHLH* members. Different colors and quantities in the grid indicate the number of cis-regulatory elements. The histogram on the right shows the total sum of each type of cis-regulatory element.

*bHLH* genes play vital roles in plant growth, development, and responses to various abiotic stresses. To further investigate the specific functions of the *DlbHLH* gene, we used PlantCARE to identify and analyze 3,122 potential cis-elements in the 2,000-bp promoter region upstream of the *DlbHLH* start codon. The frequency of light response elements was the highest among all *DlbHLH* promoters, followed by that of hormone-responsive elements, stress-responsive elements, and growth and development response elements. The hormone-responsive elements included jasmonic acid response, abscisic acid response, gibberellin response, salicylic acid response, and auxin response. The stress-responsive elements included drought-inducible elements and low-temperature response elements (**Figure 5**). Subsequently, we used Excel spreadsheets to plot histograms representing the total sum of each type of cis-regulatory element. In conclusion, *DlbHLH* expression was closely related to light and stress conditions. *DlbHLH* members perform vital functions in photoperiod, circadian rhythms, hormones, and stress pathways, demonstrating the functional diversity of *DlbHLH*.

### 3.7 Expression patterns of *DlbHlH* in different tissues and at different developmental stages

**Figure 6:**
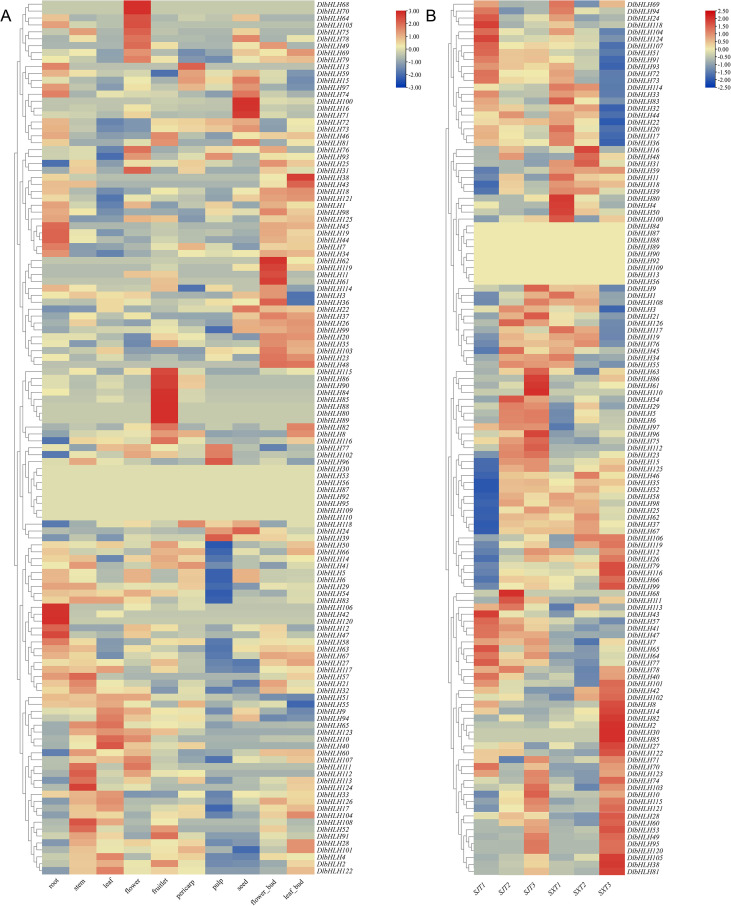
Expression patterns of *DlbHLH* genes in different tissues **(A)** and at different flowering stages **(B)**. Color gradients (red/yellow/blue) indicate expression levels (from high to low).

To investigate the potential biological functions of *DlbHLH* genes, we analyzed their expression patterns in different tissues of longan (roots, stems, leaves, flowers, fruits, etc.) (**Supplementary Table 7**) and various floral stages of two different cultivars: SX (flowers once a year) and SJ (blossoms several times a year) based on existing transcriptomic data. The floral stages included T1 (dormant period), T2 (flower primordial stage), and T3 (formation of floral organs). As shown in **Figure 6A**, *DlbHLH* members exhibited distinct tissue-specific expression patterns, suggesting extensive functional differentiation during longan tree growth and development. Based on the evident expression characteristics, these genes can be roughly divided into the following functional groups: 1) root-dominant expression genes, including *DlbHLH106*, *DlbHLH42*, *DlbHLH120*, etc., which might participate in root development or stress responses by underground parts; 2) dominant expression genes in flowering parts, such as *DlbHLH68* and *DlbHLH70*, which had the highest expression levels in flower tissues, strongly suggesting that they played specific roles in reproductive processes such as flower organ formation or pollen development; 3) fruit and seed development–related genes, a significant gene cluster that included *DlbHLH115*, *DlbHLH86*, *DlbHLH90*, *DlbHLH84*, *DlbHLH85*, *DlbHLH88*, *DlbHLH80*, and *DlbHLH89*, which were all highly expressed in the fruit body; 4) *DlbHLH100*, *DlbHLH16*, *DlbHLH71*, and other genes actively expressed in seeds and may be related to fruit quality formation or seed development regulation; 5) *DlbHLH62*, *DlbHLH119*, *DlbHLH11*, and *DlbHLH61*, all of which were expressed highly in leaf buds; and 6) *DlbHLH38* and *DlbHLH43*, which were highly expressed in flower buds, making them key candidate genes regulating the differentiation of buds or flower buds. In addition to these six categories of genes, there were a number of low expression or non-expressed genes, such as *DlbHLH30*, *DlbHLH53*, and *DlbHLH56*, which showed no significant expression in any of the tissues tested. The functions of these genes may be activated only under specific stress conditions or during extremely brief developmental windows.

Expression analysis of the two varieties, SX and SJ, at three flowering stages (**Figure 6B**) further revealed the dynamic regulatory role of the *DlbHLH* gene in flower induction. In the SJ cultivar, 23 genes, including *DlbHLH35*, *DlbHLH37*, and *DlbHLH46*, exhibited significantly upregulated expression during the initial floral initiation phase (SJT1-SJT2); *DlbHLH8*, *DlbHLH24*, and *DlbHLH123* exhibited downregulated expression at the same stage, with *DlbHLH8* being significantly downregulated by 7.49-fold (**Supplementary Table 8**). In the later stage (SJT2-SJT3), 11 genes, including *DlbHLH74*, *DlbHLH81*, and *DlbHLH95*, were upregulated, whereas *DlbHLH42* and *DlbHLH82* were downregulated. In the SX cultivar, seven genes, including *DlbHLH119*, *DlbHLH106*, and *DlbHLH48*, were upregulated during the SXT1-SXT2 stage; seven other genes, including *DlbHLH24*, *DlbHLH38*, and *DlbHLH40*, were downregulated. Among the numerous genes, the expression pattern of *DlbHLH8* was particularly notable. In the continuously flowering SJ cultivar, *DlbHLH8* showed a striking 7.49-fold downregulation during the critical initial transition from the dormant period (T1) to the flower primordium stage (T2). Conversely, in the once-a-year flowering SX cultivar, *DlbHLH8* was sharply upregulated (27.45-fold) during the later stage of floral organ formation (T2 to T3). This dramatic and cultivar-specific expression shift during pivotal developmental windows strongly suggests that *DlbHLH8* plays a complex yet potentially crucial role in the regulation of longan flowering. Based on its significant differential expression, we selected this gene and its cognate protein for subsequent subcellular localization and transgenic functional validation analyses.

### 3.8 Subcellular localization analysis of *DlbHLH8*

**Figure 7:**
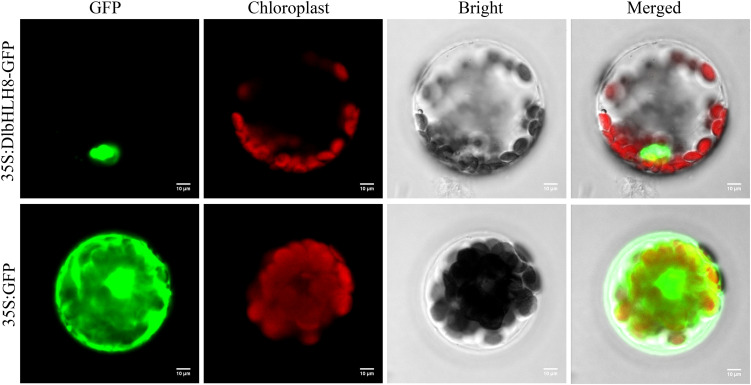
Subcellular localization of DlbHLH8-GFP fusion protein in *Arabidopsis* protoplasts. Green fluorescent protein (GFP) and chloroplast (CP) fluorescence signals were observed at a wavelength of 480 nm in protoplasts. Scale bar, 10 μm.

To investigate the subcellular localization of the DlbHLH8 protein, we first performed predictions using the WoLF PSORT online tool, which demonstrated that the protein was localized to the nucleus, consistent with the typical distribution characteristics of transcription factors. To validate this prediction, we further constructed a *35S*:GFP-DlbHLH8 fusion expression vector and transiently expressed it in *Arabidopsis* protoplasts. Confocal microscopy clearly demonstrated that the DlbHLH8 protein was specifically localized to the nucleus (**Figure 7**); this observation served as cytological confirmation of its transcriptional regulatory factor activity.

### 3.9 Overexpression of *DlbHLH8* promotes flowering in *Arabidopsis thaliana*

**Figure 8:**
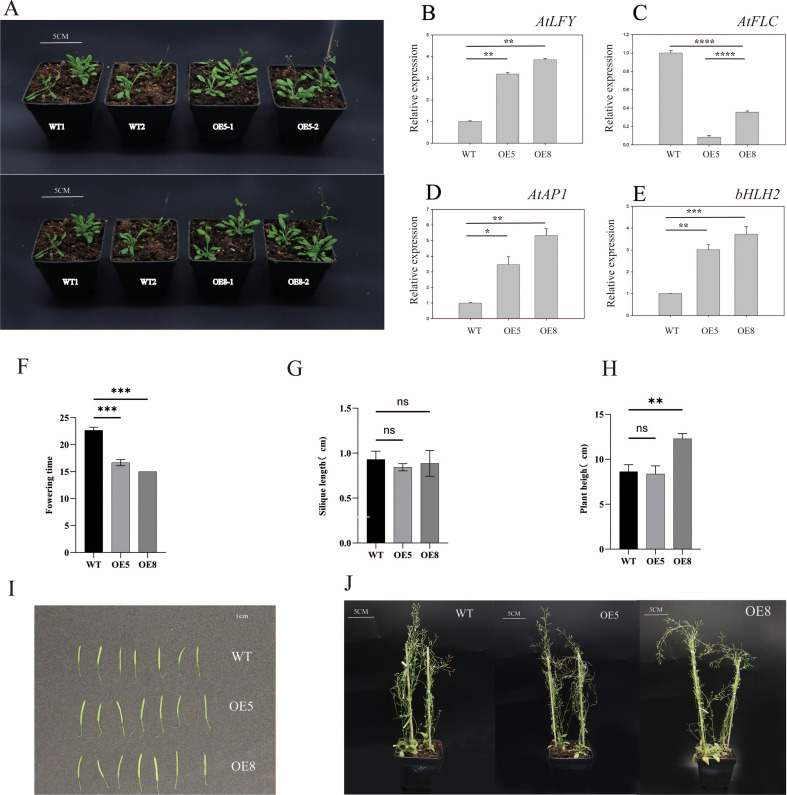
Analysis of *DlbHLH8* transgene overexpression. Comparative chart of flowering time between wild-type (WT) *Arabidopsis* and transgenic *Arabidopsis* **(A)**. Expression levels of *AtAP1*, *AtLFY*, *AtFLC*, and *bHLH2* in transgenic and wild-type *Arabidopsis* lines were measured via RT-qPCR **(B–E)**. Asterisks denote statistically significant differences. Data are presented as the mean ± standard deviation (SD) from three biological replicates for RT-qPCR and from at least three individual plants for phenotypic traits. Asterisks denote statistically significant differences compared with the wild-type control (ns p > 0.05 (No significant), *p < 0.05, **p < 0.01, ***p < 0.001, ****p < 0.0001). Days to flowering in *Arabidopsis*, silique length, and plant height statistical analysis **(F–H)**. Biomass comparison chart **(I, J)**.

To investigate the biological function of *DlbHLH8* in flowering time regulation, given the current difficulties in longan genetic transformation, we selected *Arabidopsis thaliana* as a model plant for heterologous overexpression functional validation. We selected T3-generation homozygous transgenic *Arabidopsis thaliana* lines (OE5 and OE8) as the genetic materials. Phenotypic observations revealed that under long-day conditions, transgenic plants displayed notably earlier flowering than wild-type plants (**Figure 8A**).

To further clarify the molecular mechanisms by which *DlbHLH8* controlled flowering time, we analyzed changes in the expression of multiple *Arabidopsis* endogenous flowering-related genes in transgenic lines. The RT-qPCR results (**Figures 8B–E**) demonstrated that in *DlbHLH8*-overexpressing lines (OE5 and OE8), the expression levels of the floral meristem identity genes *AtAP1* and *AtLFY* were significantly higher than those in the wild-type. Conversely, the expression level of the flowering repressor *AtFLC* was significantly lower in these strains than in the wild-type. These results associated *DlbHLH8* overexpression with the transcriptional modulation of key *Arabidopsis* flowering time regulators, consistent with the observed early-flowering phenotype. These results suggest that *DlbHLH8* participates in the molecular pathway regulating the transition from vegetative to reproductive growth in plants, potentially through pathways involving the floral integrator genes *AtAP1*, *AtLFY*, and *AtFLC*. However, the precise regulatory mechanisms, including whether this regulation is direct or indirect, require further analysis and validation.

OE8 and OE5 flowers were observed at 15 and 17 d post-transplantation, respectively (**Supplementary Table 9**), whereas wild-type plants required 22–24 d (**Figure 8F**). The average lengths of wild-type, transgenic plant OE5, and transgenic plant OE8 siliques were 0.93 ± 0.09, 0.84 ± 0.04, and 0.89 ± 0.14 cm, respectively. There was no significant change among the three groups (**Figures 8G, I**). The average plant height was 8.37 ± 0.90 cm for OE5, 12.30 ± 0.55 cm for OE8, and 8.63 ± 0.75 cm for the wild-type, with plant height being significantly greater in OE8 than in the wild-type (**Figures 8H, J**).

In summary, the results of these experiments serve as verification of the flower-promoting function of *DlbHLH8* in *Arabidopsis thaliana*, providing important functional clues for understanding its role in the flowering of longan trees.

## 4 Discussion

The complex flowering regulation of longan, which is highly sensitive to photoperiod and environmental cues, is a key determinant of its fruit yield and quality (Jue et al., 2018b). The contrasting flowering habits of the SX variety, which flowers once a year (Jue et al., 2019; Potchanasin et al., 2009; Jue et al., 2024), and SJ variety, which can flower multiple times annually (Liu et al., 2023; Jue et al., 2022; Jia et al., 2014), provide an ideal comparative system for dissecting the molecular mechanisms underlying the floral transition in longan. In this study, we performed the first systematic genome-wide identification of the *bHLH* gene family in longan, detecting 126 members. Despite having a genome size (444 Mb) similar to that of grape (487 Mb), longan possesses more *bHLH* genes than grape (110) (VanBuren et al., 2011; Sun et al., 2025). Even when compared with tomato, which has a substantially larger genome (900 Mb) but only slightly more *bHLH* genes than longan (140) (Shi et al., 2025), it is evident that the DlbHLH family has undergone a notable species-specific expansion, likely via lineage-specific gene duplication events.

The bHLH transcription factor family represents a central regulatory hub in plants, integrating diverse signals from light, hormones, and stress to orchestrate key developmental transitions (Das et al., 2025; Liang et al., 2017; Li et al., 2016; Gao et al., 2024). In the context of flowering, *bHLH* genes have been functionally implicated in several species, such as *OsbHLH1* in rice, which regulates heading date (Wang et al., 2003). Our phylogenetic analysis placed the 126 DlbHLH proteins into 26 of the 27 recognized subfamilies defined in *Arabidopsis*. Notably, several *DlbHLH* genes clustered within subfamilies containing well-characterized flowering regulators, including Phytochrome Interacting Factors (PIFs). This evolutionary conservation strongly supports the hypothesis that certain *DlbHLH* members are primed to participate in the photoperiodic regulation of flowering in longan.

Further supporting this hypothesis, the *DlbHLH* genes exhibited a non-random chromosomal distribution, with Chr 4 harboring the most members (16) and Chr 3 harboring only one. Genes within the same subfamily generally shared similar exon-intron structures and motif compositions, indicating strong evolutionary constraints on their protein architectures. This intra-subfamily conservation aligns with the notion that the key integrators of flowering pathways, such as *FLOWERING LOCUS T* (*FT*) and *CONSTANS* (*CO*), typically possess relatively simple and conserved gene structures in plants (Wickland and Hanzawa, 2015). The systematic analysis of the *Arabidopsis thaliana* bHLH family by Toledo-Ortiz et al. identified an “orphan” subfamily of 21 members that were distantly related to other bHLH proteins (Toledo-Ortiz et al., 2003). Notably, our analysis revealed that longan possessed homologs in several conserved flowering-related subfamilies but lacked any member in the orphans(2) subfamily, further underscoring the evolutionary conservation of the flowering-related bHLH repertoire in longan.

Collinearity analysis revealed the evolutionary dynamics of the bHLH family in longan. We found that its expansion primarily relied on segmental duplication, which is consistent with observations in species such as *Phyllostachys edulis*, indicating that this mechanism was conserved in the evolution of bHLH families in monocotyledonous and dicotyledonous plants (Xiu-Rong et al., 2019). A total of 152 orthologous *bHLH* genes were identified between longan and its closely related species lychee, significantly higher than the 105 pairs observed in *Arabidopsis*. Using phylogenomic analysis, Hu et al. demonstrated that longan and lychee belonged to the same family, Sapindaceae, with extremely close genetic relationships and high genomic collinearity (Hu et al., 2022). The functions of orthologs are often conserved. Therefore, we hypothesized that *DlbHLH8*, the longan ortholog of longan in lychee, played a similar role in this process, making it a highly promising candidate gene for studying the flowering mechanism of longan. Furthermore, Ka/Ks analysis of the 23 high-confidence segmentally duplicated *DlbHLH* gene pairs revealed that all exhibited Ka/Ks ratios significantly less than 1 (**Table 1**), indicating that strong purifying selection acted to maintain the functional integrity of these duplicated genes. This evolutionary constraint is consistent with the high intra-subfamily conservation of gene structure and protein motifs observed in our study; it aligns with similar patterns of purifying selection reported in other plant bHLH families, such as those in barley (Ke et al., 2020), *Pueraria lobata* (Xiao et al., 2023), and *Rosa roxburghii* (Li et al., 2026). These findings collectively reinforce the notion that the expansion of the DlbHLH family through segmental duplication is accompanied by functional retention rather than by rapid neofunctionalization.

Promoter region analysis showed that the promoter region of the *DlbHLH* gene was enriched with multiple hormone and stress response elements, consistent with the results reported by Musa et al. in common bean (Kavas et al., 2016). Specifically, the predominance of light-responsive elements across the promoters of many *DlbHLH* genes is particularly noteworthy. Longan flowering is highly sensitive to photoperiod; this enrichment strongly implies that a subset of DlbHLH proteins function as key integrators of light signals to regulate the floral transition. This condition is reminiscent of the well-established role of PIFs, a subgroup of bHLH transcription factors, in *Arabidopsis thaliana*. PIFs interact with phytochromes to mediate light-regulated growth and development, including flowering time modulation. The presence of numerous *DlbHLH* genes in the same phylogenetic subfamilies as PIFs suggests that a similar light-signaling hub operates in longan. Future studies should focus on identifying which specific *DlbHLH* members respond to photoperiodic cues and how they interface with the conserved flowering time machinery, such as the CO–FT module, to control the unique flowering habits of different longan cultivars. In contrast to the focus on heat stress response elements in the longan *bZIP* gene family (Szulc et al., 2020), we detected a superior abundance of photoresponsive elements in *DlbHLH* promoters, highlighting the functional specificity of different transcription factor families in signal transduction networks (Wang et al., 2026).

Analysis of tissue-specific expression showed that the *DlbHLH* gene had obvious functional differentiation in different longan tissues. For example, genes such as *DlbHLH106* and *DlbHLH42* were highly expressed in the roots, whereas *DlbHLH68* and *DlbHLH70* exhibited tissue-specific expression in the floral parts, suggesting their potential involvement in tissue-specific developmental regulation. This finding aligns with those of Li et al. (2025), which showed that *bHLH* genes in rice regulated plant growth and development through tissue-specific expression patterns (Li et al., 2025). In the analysis of flowering time expression, we discovered that *DlbHLH8* expression was markedly reduced from T1 to T2. In SJ, *DlbHLH8* transcript levels dropped sharply as the plant transitioned from dormancy to floral initiation. Elevated *DlbHLH8* expression in the dormant bud may play a role in maintaining the vegetative state or repressing precocious flowering; its downregulation is a prerequisite for the floral transition to proceed. Alternatively, the function of *DlbHLH8* may be highly dosage- and context-dependent; therefore, its precise temporal regulation, rather than its absolute expression level, is critical for proper floral induction. The contrasting upregulation of *DlbHLH8* during the later stages of flower development in SX further supports the notion that this gene plays distinct roles at different phases of the reproductive process. Future experiments modulating *DlbHLH8* expression at specific developmental stages in longan will be necessary to resolve this complexity.

Subsequent functional validation experiments demonstrated that *DlbHLH8* overexpression promoted *Arabidopsis* flowering. In transgenic *Arabidopsis*, *AtAP1*, *AtLFY*, and *AtFLC* were significantly expressed, with *AtAP1* and *AtLFY* displaying marked upregulation. This finding is consistent with the conclusions drawn in two previous studies (Zhang et al., 2024; Xu et al., 2022): first, *MdGAMYB* overexpression can promote the expedited flowering of transgenic *Arabidopsis thaliana* and tomatoes by upregulating the expression of the flowering-promoting gene *LFY*; second, *PfFT1* can promote early flowering in plants by regulating the expression of flower-related genes *AtAP1* and *AtLFY. AtFLC* expression was significantly downregulated, which is consistent with reported research conclusions: MYC2 regulates photomorphogenesis growth and flowering time by mediating FLC, CPL2, and CPL3, which play redundant roles in the activation of FLC and regulation of flowering time in *Arabidopsis thaliana*, and the molecular switch of activation at the flowering site C determines flowering time in *Arabidopsis thaliana*. These results prove that *AtFLC* inhibits flowering (Chakraborty et al., 2025; Zhang and Shen, 2022; Shen et al., 2022). The results of our study further revealed that the *DlbHLH*8 gene may promote flowering through interactions among *AtAP1*, *AtLFY*, and *AtFLC*. Lastly, the nuclear localization pattern of *DlbHLH8* is consistent with reports concerning multiple *bHLH* proteins involved in light signaling or developmental regulation in plants such as tomato, rice, and maize (Kurt et al., 2019), all of which are localized in the nucleus, suggesting potential functional conservation. This was referenced in studies on related *bHLH* members (e.g., PIFs) in *Arabidopsis thaliana* (Xu X. et al., 2017).

This study provides a systematic identification and functional analysis of the *bHLH* gene family in longan (*Dimocarpus longan* Lour.), offering crucial genetic materials for molecular breeding. Although the function of *DlbHLH8* in flowering regulation has been elucidated, the upstream–downstream interaction network of *DlbHLH8* and its comprehensive impact on floral organ development remains a subject for further research.

## 5 Conclusion

We identified 126 *bHLH* members in longan, which were divided into 27 subfamilies. The analysis showed that the family was distributed unevenly across the genome and that its expansion was mainly propelled by segmental duplication events under strong purifying selection. Promoter analysis uncovered that most *DlbHLH* genes harbored ample cis-acting elements involved in light, hormone, and abiotic stress responses. Expression profiling further revealed that members of this family exhibited specific expression profiles in diverse tissues and during critical stages of flower induction, with *DlbHLH8* exhibiting a significant downregulation during the initial floral transition phase (T1 to T2) in the SJ cultivar, but a sharp upregulation in the later floral organ formation phase (T2 to T3) in the SX cultivar. Subcellular localization confirmed that the DlbHLH8 protein was localized in the nucleus. Functional validation results demonstrated that *DlbHLH8* overexpression in *Arabidopsis* markedly accelerated early flowering in transgenic plants and was associated with altered expression of key flowering pathway genes, including *AtAP1*, *AtLFY*, and *AtFLC*. In summary, this study is the first to systematically identify the *bHLH* gene family in longan and suggest that DlbHLH8, as a nuclear-localized transcription factor, plays a complex and likely critical role in controlling flowering time, laying a solid basis for a comprehensive analysis of the molecular mechanism of longan flower development.

## Data Availability

The datasets presented in this study can be found in online repositories. The names of the repository/repositories and accession number(s) can be found in the article/**Supplementary Material**.
